# Silent refractoriness in epilepsy care: cross-sectoral analysis of prescribing patterns, referrals and workforce capacity in Saxony

**DOI:** 10.3389/fneur.2026.1855403

**Published:** 2026-07-21

**Authors:** Thomas Mayer, Johannes Lemke, Felix Von Podewils, Brigitte Scheid, Torsten Kraya, Susanne Hallmeyer-Elgner, Joseph Classen, Karel Kostev, Sonya C. Faber, Adam Strzelczyk

**Affiliations:** 1 Epilepsiezentrum Kleinwachau Gemeinnutzige GmbH, Radeberg, Germany; 2 Universitatsklinikum Leipzig Institut fur Humangenetik, Leipzig, Germany; 3 Department of Neurology, Universitatsmedizin Greifswald Klinik und Poliklinik fur Neurologie, Greifswald, Germany; 4 Neurologie.Psychiatrie Practice im Königsbau, Leipzig, Germany; 5 St. George Hospital Leipzig, Leipzig, Germany; 6 Neurologische Praxis, Dresden, Germany; 7 Department of Neurology, Leipzig University Medical Center, Leipzig, Germany; 8 IQVIA Commercial GmbH and Co OHG, Frankfurt, Germany; 9 IQVIA Germany, Real World Solutions, Frankfurt am Main, Germany; 10 Angelini Pharma Deutschland GmbH, Munich, Germany; 11 Epilepsy Center Frankfurt RhineMain, Department of Neurology, Goethe University Frankfurt, Frankfurt am Main, Germany

**Keywords:** ASM, demographics, epilepsy, prescribing, Saxony

## Abstract

**Background:**

Epilepsy care in Germany is structured through a delegated outpatient model in which long-term management is primarily delivered by office-based neurologists and general practitioners, while specialized epilepsy centers provide escalation for complex cases. Whether this structure enables timely access to advanced therapies in high-burden regions remains unclear. Objective: To evaluate structural disparities in prescribing patterns, referral behavior, and workforce distribution in Saxony, a federal state with above-average epilepsy prevalence and treatment intensity.

**Methods:**

We conducted a cross-sectoral analysis integrating IQVIA LRx® prescription data (2024), Disease Analyzer prevalence estimates, and anonymized outpatient referral data from the state’s only DGfE-certified epilepsy center (Kleinwachau). Utilization was assessed using Days of Therapy (DOT), with a focus on levetiracetam (LEV) as a proxy for focal epilepsy care. Workforce distribution was estimated using Bundesärztekammer data. Findings were contextualized through a multidisciplinary expert panel.

**Results:**

Despite high treatment intensity, substantial gaps in escalation were observed. While approximately 36% of patients met a pharmacological proxy for drug resistance, only a minority accessed specialized center care. Prescribing patterns showed a persistent preference for LEV across all sectors, with marked regional variation in LEV: lamotrigine ratios that aligned with levels of specialization and proximity to the epilepsy center. Adoption of newer antiseizure medications was concentrated in specialized hubs. Referral activity remained uneven, with several regions demonstrating minimal connectivity to formal escalation pathways.

**Conclusion:**

Epilepsy care in Saxony is characterized by “silent refractoriness,” in which therapy-resistant patients remain in decentralized care without systematic progression to specialized evaluation. Addressing this gap requires implementation of standardized referral criteria, strengthened cross-sectoral networks, and regional accountability mechanisms. These findings provide a scalable framework for aligning decentralized care systems with evidence-based escalation pathways.

## 1 Introduction

Epilepsy represents one of the most prevalent neurological conditions in Germany, affecting approximately 1% of the population. Despite the availability of numerous newer therapeutic options, approximately 30 to 40% of patients have a drug-resistant epilepsy (1). For this cohort, specialized evaluation at a tertiary epilepsy center is the established standard of care to explore surgical or advanced pharmacological interventions. However, historical data suggests that many patients face significant delays before accessing these centers, often remaining in secondary care for years despite persisting seizures (2).

In the German healthcare system, the outpatient sector serves as the primary gateway for longitudinal epilepsy management. Care is structured through a delegated model in which office-based neurologists and general practitioners provide the majority of long-term treatment, while hospital-based specialists contribute expertise in cases of drug-resistant epilepsy. The successful implementation of evidence-based guidelines therefore depends on effective coordination across sectors, particularly for therapies requiring prolonged titration, rational polytherapy, and close monitoring of drug interactions. When this coordination is inconsistent, maintaining guideline-concordant care in routine practice becomes more challenging, especially for patients with therapy-resistant or multi-morbid profiles. In this context, patient outcomes are shaped not only by the availability of pharmacological options, but also by the continuity and integration of the care framework (3).

We integrated external morbidity data from the BARMER Institute for Health Systems Research (bifg, 2023) to provide a closer look at patient care in epilepsy on a regional level. These data reveal that the Federal State of Saxony occupies a unique position in the German healthcare landscape. While the national average prevalence for epilepsy is 12.87 per 1,000 inhabitants, Saxony reports a significantly higher rate of 17.07 per 1,000 and ranks among the federal states with the highest epilepsy associated morbidity burdens. In addition to the elevated prevalence, BARMER 2023 data indicate that Saxony is also characterized by a higher-than-average medication burden per affected person, reflected in increased Defined Daily Dose (DDD) exposure and a greater diversity of prescribed active substances compared to the national mean. The elevations above the federal baseline makes the identification of structural treatment gaps a matter of significant public health urgency and suggest that epilepsy care in Saxony has not only one of the highest medication burdens but is also pharmacologically complex at the patient level This complexity is highly relevant to clinical outcomes, as the International League Against Epilepsy (ILAE), defines drug resistant epilepsy as the failure of two appropriately chosen and tolerated ASMs (4).

These external benchmarks provide the context against which new statewide clinical referral data and prescribing volumes can be measured. Together, the combination of higher morbidity, increased prevalence and elevated medication burden underscores the importance of examining how treatment pathways are structured and how escalation to specialized care is implemented within the region.

While the BARMER data confirm a disproportionately large and pharmacologically complex patient population, it remains unclear to what extent this burden is accompanied by timely access to specialized escalation pathways and newer therapeutic options. Despite the approval of more efficacious ASMs such as fenfluramine and cenobamate, real-world evidence suggests that breakthrough treatments are often adopted slowly in routine care (5). In Germany, the structural division between hospital-based and office-based sectors, each operating under different prescribing frameworks, may further influence how and where advanced therapies are initiated. As a result, regional variation in treatment patterns may reflect not only clinical need, but also differences in system organization.

This study examines the epilepsy care landscape in the German Federal State of Saxony between 2022 and 2024 to investigate these structural tensions. By utilizing regional prescribing volumes and spatial referral data, we analyze the relationship between physician specialty, geographic location, and adherence to international treatment standards.

The data was evaluated from a clinical and scientific standpoint with health care providers and epilepsy experts who discussed and assessed the systemic hurdles that determine patient access and suggested solutions. Through this combined analytical and expert-led approach, we seek to identify the specific barriers in the care pathway, provide Saxony specific recommendations, and establish a basis for longitudinal benchmarking in a representative German federal state (Tables 1, 2).

**Table 1:** Distribution of referring healthcare providers and referred epilepsy patients from Saxony to the specialized center in 2022 and 2024.

TYPE of physician	Referring HCPs from Sachsen 2022	Referring HCPs from Sachsen 2024	Referred Patients from Sachsen 2022	Referred Patients from Sachsen 2024
AMB Neurologists/AMB Kleinwachau	12	5	69	516
AMB Kleinwachau Dresden	0	4	0	296
Neurologists (NEU + MVZ)	100	125	296	422
Pediatricians	129	98	207	234
GPs, Psychologists, Others	175	142	213	522
Total	416	374	786	1990

In the German healthcare system, specialized hospital outpatient clinics typically operate under a referral requirement and do not serve as primary sources of external referrals to other centers. The 812 patients recorded for the Kleinwachau and Dresden AMB sites primarily represent internally managed specialized cases and institutional transfers. The high patient volume relative to the small core of 9 specialists (a ratio of approximately 90:1) reflects the centralization of pharmacological leadership, distinguishing it from referrals (max 15/year) from office-based neurologists.

**Table 2:** Systemic hurdles and suggested solutions.

Topic	Identified hurdle	Proposed strategic solution	Accountability/ownership
Acute to chronic care	*Discharge gap*: Patients lost to follow-up after ER visit; overreliance on LEV, underreliance on LTG	Standardized discharge management (Entlassmanagement) with mandatory neurologist referral.	Hospital management and quality assurance departments
Referral structures	*Knowledge gap*: Lack of clarity among neurologists/APIs on treatment failure and ASM use	Development of a “Red Flag” catalog to identify insufficiently treated patients.	Regional Neurological Societies (e.g., DGfE/DGN Saxony chapters)
Referral structures	*Expectation gap*: Inertia in referral of Drug-resistant epilepsy patients; lack of perceived benefit from referral, and an absence of regional benchmarking, leaving providers unaware of personal and local suboptimal care	Publication of this study in the Sächsisches Ärzteblatt with clear referral criteria and longitudinal benchmarking (every 2 years) to provide a measurable comparison between outpatient and specialized center metrics.	Kassenärztliche Vereinigung Sachsen (KVS) and Hospital Lead Physicians
Patient advocacy	*Information gap*: Patients are unaware of the specialized care pathways available.	Patient Empowerment: Awareness campaigns, public ambassadors, and distribution of structural flyers.	Patient Support Groups (e.g., Deutsche Epilepsievereinigung)
Guidelines and ASM	*Clinical complexity*: Epilepsy treatment is highly individual and heterogeneous.	Establishment of a communication network in Saxony; dedicated contact points for office-based doctors.	University Epilepsy Centers with Kleinwachau (Consortium of Regional Clinics)
Professional education	*Skill decay*: Inconsistent expertise in complex EEG analysis and case management.	Targeted training for neurologists and APIs, specifically focusing on case discussions and EEG.	State Medical Chamber (Landesärztekammer Sachsen)

## 2 Methods

### 2.1 Estimation of regional physician workforce

To contextualize prescribing patterns, the regional neurological workforce in Saxony was estimated using data from the 2023 Federal Medical Register of the German Medical Association (Bundesärztekammer, BÄK, Table 3). While the BÄK provides the absolute total of professionally active neurologists for each federal state (*n* = 601 for Saxony), detailed sub-sectoral breakdowns (e.g., employment status within specific sub-specialties) are often only provided at the national level (6).

**Table 3:** Estimated subsectoral distribution and regional breakdown of the neurological workforce in Saxony.

Neurologists	Total	Outpatient (Ambulant)	Hospital (stationär)	Administrative (Behörden)	Other (Sonstige)	Estimated office-based within outpatient (Niedergelassene unter Ambulant)
Saxony (Sachsen)	601	245	345	6	5	157

Bundesärztekammer (6).

To determine the regional distribution, we applied a top-down attribution methodology. We utilized national fixed points, defined as the verified proportional breakdown of the 9,636 neurologists across Germany, and applied these ratios to the Saxon total (6). This allows for a high-fidelity estimation of the local workforce structure where state-specific granular data was unavailable.

### 2.2 Definition of German physician categories

The neurological workforce in Saxony is distributed across two primary sectors, each defined by distinct regulatory mandates regarding patient care and prescribing. The Inpatient Sector represents the numerical majority of neurologists (*n* = 345; 57%). While these clinicians manage acute stabilization, their impact on longitudinal outpatient data is structurally limited. In the German healthcare system, hospitals typically bridge the immediate post-discharge period via direct dispensing of minimal medication quantities (e.g., a 3-day supply) from internal ward stock or by issuing short-term Discharge Management prescriptions. Because direct dispensing is managed through internal hospital procurement and discharge prescriptions are limited to a 7-day “bridge,” these units do not appear in retail pharmacy prescriptions or longitudinal utilization datasets.

An exception is the Hospital Outpatient (AMB) category, which includes specialized university clinics, institutional clinics, and physicians with specific state authorization. These entities possess a mandate for GKV-reimbursed (statutory health insurance) outpatient care but represent a high-specialization minority of the total prescribing volume. Consequently, chronic ASM management and longitudinal patient steering are primarily centered in the Outpatient Sector (*n* = 245). These physicians operate within the GKV framework and hold the primary authority for long-term prescribing. This sector is further categorized by employment status: self-employed specialists in independent office-based practices (*n* = 157) and employed specialists practicing within Medical Care Centers (MVZ) (*n* = 88). These MVZs function as multidisciplinary community polyclinics and represent an increasingly prevalent model of integrated outpatient care. By focusing on these outpatient sectors, our analysis captures the stable, longitudinal treatment pathways that define focal epilepsy care in the region.

### 2.3 Data source and patient population

The prescribing data for this study were obtained from the IQVIA LRx® (Longitudinal Prescription Data) database for the year 2024. This database captures anonymized, pharmacy-based prescription records across Germany. The patient cohort for this study was defined based on established regional prevalence data for Saxony which is derived from the IQVIA™ LRx and Disease Analyzer databases and provided on state levels in the publication of Strzelczyk et al. (7). For Saxony, a cohort of 31,000 patients was assessed.

For a national data analysis of different prescriber specialties, relevant for care provisioning in epilepsy, IQVIA™ Pharmascope® Docsplit has been leveraged to split relevant metrics and analysis by specialty groups. The analysis included prescriptions from specialty groups in office-based retail setting such as Neurologists and General Practitioners (GPs) as well as prescriptions filed by outpatient clinics (Ambulanzen) dispensed by retail pharmacies. The underlying coverage of prescriptions of IQVIA LRx® and IQVIA Pharmascope® is similar with approximately 80% of all prescriptions reimbursed by statutory health insurance in Germany (7, 8). This study combines multiple complementary datasets. Where available, the most recent data (2024) were used for cross-sectional analyses, while referral trends were examined longitudinally between 2022 and 2024.

### 2.4 Quantitative metrics: days of therapy utilization metrics

Utilization was quantified using Days of Therapy (DOT) as described by Strzelczyk et al. (9). One DOT represents a single day of treatment with the specified medication, independent of dosage. The DOT metric provides a high-fidelity representation of actual patient exposure, as it calculates the number of days a patient is covered by a medication regardless of the specific milligram dosage. This is particularly relevant in epilepsy, where dosages are highly individualized.

Levetiracetam (LEV) and lamotrigine (LTG) define the epilepsy treatment pathway in the German healthcare system (9). National prescription data demonstrate that LEV represents the dominant first-line ASM, prescribed to 76.6% of patients at treatment initiation, indicating its role as a broadly accessible baseline therapy across provider types (9). While differences between general practitioners and neurologists are observable for other standard ASMs such as LTG, newer ASMs remain markedly less utilized. This distribution suggests that the use of third-generation ASMs is not part of routine baseline care but reflects treatment escalation and specialist involvement, supporting its use as a proxy for the level of epilepsy care; we estimate that this methodology captures approximately 85% of the relevant focal epilepsy population. Therefore, to evaluate the regional focal epilepsy treatment pathway within a single state, the analysis focused on LEV utilization, measured DOT in 11 districts within Saxony. This agent-specific approach was chosen to minimize data noise from broad-spectrum medications (e.g., valproate) frequently utilized in non-epilepsy indications.

### 2.5 National data integration

This analysis incorporates population-based diagnostic and prescription data from the IQVIA database suite to provide a representative view of the German treatment landscape.

#### 2.5.1 Data sources and prevalence

Epidemiological prevalence was derived from the IQVIA Disease Analyzer (DA) database, which compiles anonymous diagnostic records from approximately 3,000 outpatient practices across Germany. Patients were identified using the ICD-10 code G40. This database allows for a direct assessment of the treated patient population and serves as the foundation for calculating regional disease density.

#### 2.5.2 Refractoriness and titration patterns

Prescription trends and medication changes were analyzed using the IQVIA Longitudinal Prescriptions (LRx) database, which captures approximately 80% of all prescriptions reimbursed by statutory health insurance in Germany. To identify the population at risk for suboptimal care, the study utilized a pharmacological proxy for drug-resistant epilepsy: Controlled/Responsive: Patients receiving only one or two distinct antiseizure medication (ASM) regimens. Refractory Proxy: Patients receiving three or more distinct ASM regimens, following established methodology to approximate the ILAE definition of pharmacoresistance (4, 10).

A regimen was defined as any unique combination of ASMs, with a change recorded upon the addition, removal, or substitution of a substance. The databases contain solely anonymous data and do not identify any personal information, in compliance with §3 Abs. 6 of the German Federal Data Protection Act (17). Identification of individual patients was not possible; therefore, informed consent was not required. The authors obtained the necessary permissions to use the data from both databases.

### 2.6 Statistical approach

The analysis relies on comprehensive national datasets from the IQVIA LRx®, Pharmascope® Docsplit and Disease Analyzer sources, capturing the vast majority of statutory health insurance activity and a representative cross section of German outpatient care. Due to the massive scale of these cohorts, traditional *p* values and formal hypothesis testing were omitted. In such large datasets, even negligible variations achieve statistical significance without necessarily reflecting a meaningful clinical or systemic shift.

Instead, the study adopts a descriptive approach to maintain focus on clinical relevance. Results are presented through absolute proportions and regional distributions to highlight real world patient flows. This method ensures that the evaluation remains centered on the structural alignment of the care pathway and the practical identification of referral gaps rather than abstract mathematical thresholds.

### 2.7 Specialized center referral analysis

To supplement the regional pharmacy-based dataset, we conducted a secondary analysis of outpatient billing data from the only DGfE certified Epilepsy Center in Saxony (Kleinwachau, Radeberg, Germany). The analysis was based on anonymized KVDT (KV-Datentransfer) datasets, the standardized electronic billing format for the German Association of Statutory Health Insurance Physicians (KBV), and utilized anonymized outpatient billing records from the Kleinwachau Saxon Epilepsy Center covering the full calendar year of 2024 (Q1–Q4).

Referral pathways were analyzed using specific billing field identifiers to determine physician attribution (e.g., General Practitioner vs. Neurologist). This utilized the Specialty Code (Field 4,220) and clinic/physician identifiers (BSNR Field 4,218/LANR Field 4,242). Patient postal codes were used to evaluate the geographic reach of the center within Saxony.

All data were provided in a fully anonymized format prior to analysis. As this study involved the retrospective analysis of de-identified billing records for health services research purposes, and no individual patient can be re-identified from the resulting group statistics, it aligns with standard German guidelines for secondary data use. No intervention was performed, and no personal identifying information (PII) was accessed by the research team.

### 2.8 Assessment of scientific leadership and publishing in epilepsy

To assess the academic positioning of epilepsy care within the state, a scoping scientometric analysis was performed using the PubMed database. The search strategy targeted epilepsy related research output from the two university clinics in Saxony, specifically filtering for affiliations located in Dresden and Leipzig. Publications were categorized by their focus on “epilepsy,” distinguishing it from other neurologic conditions, such as “Parkinson” as well as “stroke.” This analysis aimed to determine the extent of institutional investment in epilepsy-related research, which may potentially influence regional referral networks.

### 2.9 Workshop design and consensus building

The recommendations provided in this study were derived utilizing a qualitative, expert-led workshop format to identify barriers to epilepsy care. All members of the workshop are also authors on this publication. Following a presentation of current regional treatment data, a multidisciplinary panel engaged in structured brainstorming sessions. Potential solutions were iteratively refined until a consensus was reached on the six primary domains of intervention: acute to chronic care, referral structures, patient empowerment, medical education, clinical networking, and guideline adherence.

Finally, the term “Neurologist” in this study aggregates two BÄK categories: Neurology and Nervenarzt (Neurology and Psychiatry). Both hold full neurological qualifications and are authorized to treat epilepsy.

## 3 Results

### 3.1 Epidemiologic context and distribution of epilepsy care in Saxony

**Figure 1:**
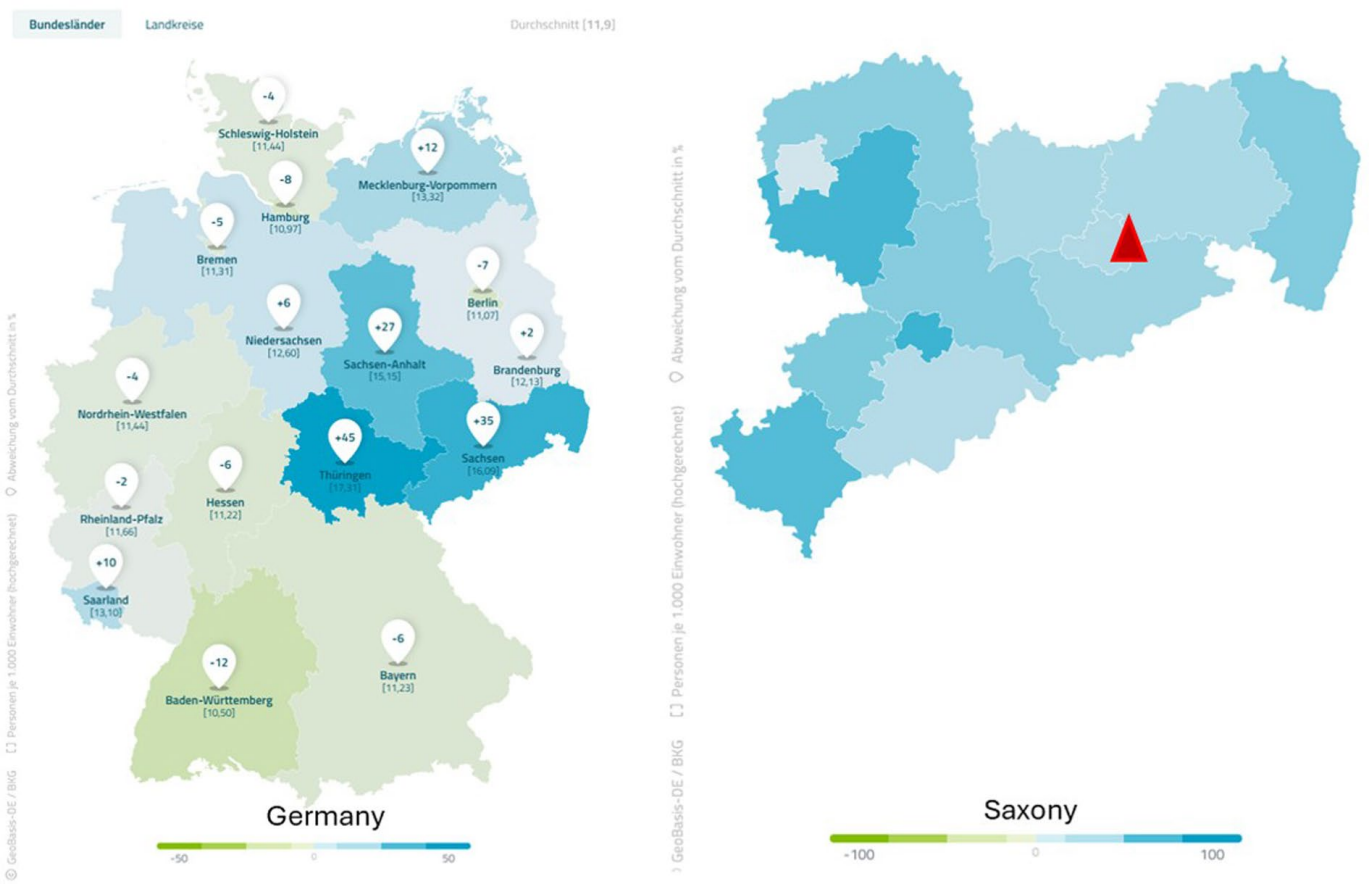
BARMER Institute for Health Systems Research (bifg) Morbidity Analysis of the Saxon Epilepsy Landscape 2023. Adapted from Epilepsie: Morbiditäts-und Sozialatlas, by BARMER Institut für Gesundheitssystemforschung (14) (https://www.bifg.de/atlas/epilepsie). Copyright 2023 by bifg. Analysis of epilepsy morbidity at the federal and state levels for the 2023 reporting period. (Left) National morbidityrates by federal state, identifying Saxony as part of a high-prevalence cluster alongside Saxony-Anhalt and Thuringia. Shading indicates percentage deviation from the national average baseline (0), ranging from a negative deviation (green, -50) to a positive deviation (blue, +50). Map pins denote the exact state-level percent deviations from this national baseline. (Right) Sub-national distribution of epilepsy morbiditywithin Saxony's districts. Higher morbidity rates are concentrated in the western districts of the federal state. This geographic distribution correlates with the healthcare infrastructure, where the eastern region is served by a specialized DGfE center in Dresden. The district-level color gradient represents localized percent deviation from the baseline (0), ranging from -100 (green) to +100 (blue). Data source: BARMER Institute for Health Systems Research (bifg). Red triangle indicates location of DGfE certified center Kleinwachau.

The Results section begins with population level morbidity data to establish the epidemiological context for subsequent analyses of prescribing behavior, referral patterns and specialist capacity. Figure 1 presents national and state epilepsy associated morbidity data identifying Saxony as a high -burden region and illustrating marked geographic heterogeneity within the state.

**Figure 2:**
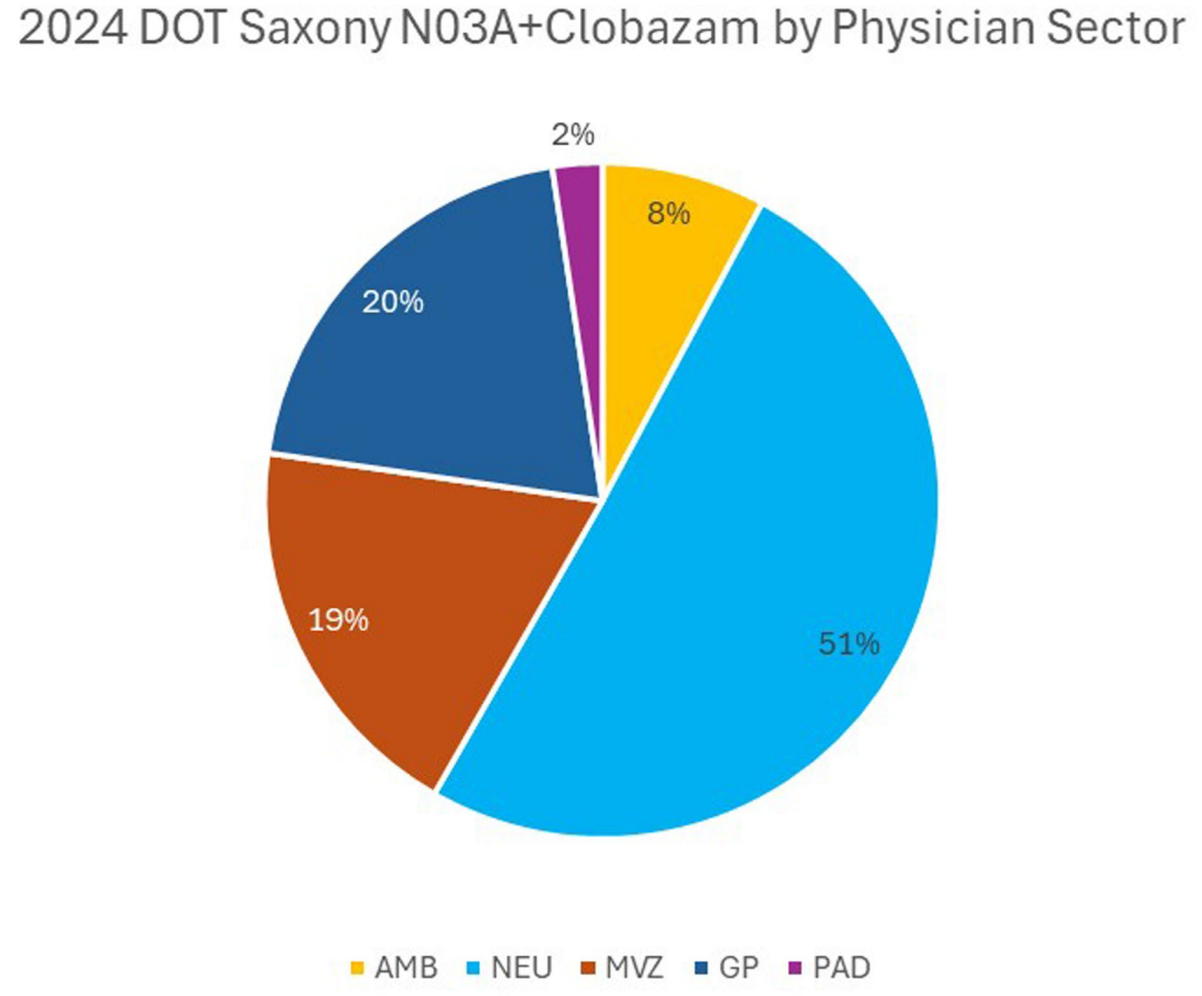
Distribution of Epilepsy treatment Volume Across Physician Sector in Saxony 2024 by DOТ Distribution of epilepsy treatment volume across physician sectors in Saxony (2024). Treatment volume was calculated using aggregate days on therapy (DOT) for all antiseizure medications classified within the ATC NO3A category, supplemented by clobazam. A total of 19,220,850 DOT were identified across the 11 Saxony districts. Neurologists accounted for 51% of treatment volume, followed by general practitioners (20%), medical care centers (MVZs; 19%), hospital outpatient clinics (AMB; 8%), and pediatric providers (2%). DOT was used as a measure of longitudinal treatment volume rather than patient counts.

The distribution of epilepsy treatment volume across the outpatient sector for 2024 is illustrated in Figure 2. Treatment volume was calculated using aggregate DOT for all ASM within the ATC N03A category plus clobazam. Consistent with analyses based on LEV, which is the dominant ASM in Saxony, the majority of treatment volume was concentrated within the outpatient neurology sector. General practitioners (GP) represent a significant share of the treatment landscape, accounting for approximately one-fifth of all prescriptions. In contrast, hospital-based outpatient departments (AMB) contribute about 8% of the total prescription volume. The majority of the prescribing burden remains concentrated within the specialized outpatient sector, divided between independent practices and medical care centers.

Observed prescribing patterns for ASMs can be interpreted as reflecting epilepsy treatment rather than off-label or non-epilepsy use. Lacosamide, which might be prescribed off-label for other indications as neuropathic pain contributes a smaller share of overall exposure and shows a consistent distribution across prescribers and regions, suggesting that its inclusion does not materially alter comparative patterns observed for epilepsy-specific treatments.

### 3.2 Regional distribution and prescriber proportions in Saxony

**Figure 3:**
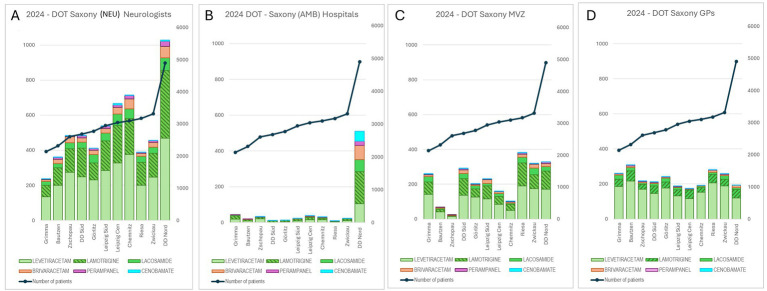
Regional Distribution and Prescriber Proportions in Saxony: Comparative Regional Analysis ASM Utilization. Regional Distribution and Prescriber Proportions in Saxony: Comparative Regional Analysis ASM Utilization. Stacked bar segments represent the total Days of Therapy (DOT) for the six primary ASMs tracked (three standard: Levetiracetam, Lamotrigine, Lacosamide; three modern Brivaracetam, Perampanel, Cenobamate) across 11 Saxon regions. Data are segmented by physician category: **(A)** Office-based Neurologists, **(B)** Specialized Hospital Outpatient Clinics (AMB) **(C)** Medical Care Centers (MVZ), and (D) GPs and Other Providers. The secondary axis (blue line) indicates the total patient volume per region for 2024 of a total 31,000 epilepsy patients. Each dot on this line represents the estimated number of epilepsy patients in the district being treated.

Figure 3 presents the use of selected established and newer antiseizure medications (ASMs) across 11 districts in Saxony covering the entire state, stratified by prescriber type and region. Utilization is ordered by the estimated epilepsy population (*N* = 31,000) derived from levetiracetam (LEV) days on therapy (DOT) (7). For most districts, prescribing volumes broadly track estimated regional need while showing variability in the relative contribution of neurologists, hospitals and general practitioners (GPs).

In the hospital sector, outpatient prescribing is nearly negligible across the state, with the singular exception of the DD Nord region. This volume is attributed to the presence of a certified DGfE (German Society for Epileptology) Epilepsy Center in Kleinwachau, which operates a specialized outpatient clinic (Ambulanz) for drug-resistant epilepsy focal cases. Excluding such specialized hubs, hospital-associated outpatient prescribing primarily reflects short-term continuation of inpatient therapy rather than long-term treatment responsibility.

An analysis of third-generation ASM utilization (Brivaracetam, Perampanel, and Cenobamate) indicates that the adoption of breakthrough treatments is broadly distributed across the Saxon healthcare landscape. While the certified center in Kleinwachau (DD-Nord) represents a high-utilization outlier for these agents, the longitudinal uptake of newer medications is predominantly driven by the office-based neurology sector. In contrast to the inpatient sector, which focuses on initial stabilization, the outpatient sector (including both office-based neurologists and MVZs) demonstrates a higher relative volume of third-generation ASM prescribing. Furthermore, GP prescribing levels in some districts of Saxony are quantitatively comparable to the volume observed in some hospital settings, highlighting the critical role of primary care in maintaining established focal epilepsy regimens.

### 3.3 Regional adoption of third generation anti-seizure medications across Saxon hospitals compared to national benchmarks

**Figure 4:**
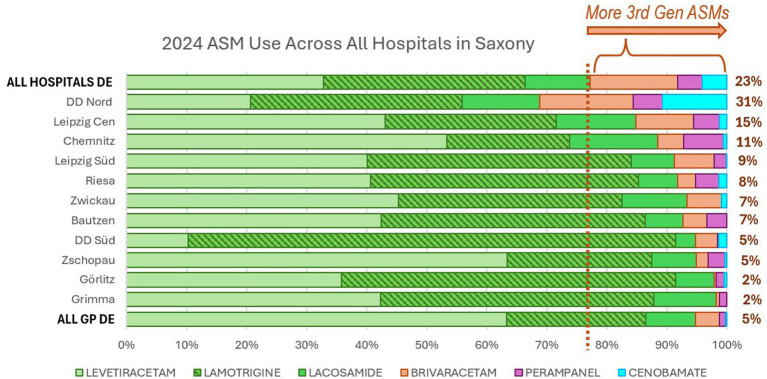
Comparative analysis of prescribing Three Third Generation ASMs across eleven Saxony hospital regions versus national benchmarks (All GP DE and All Hospitals DE). Bar segments represent the proportional utilization of six tracked ASMs, with three third generation innovative ASMs, Brivaracetam (BRV), Perampanel (PER), and Cenobamate (CNB) making up the percentage listed to the right of the bars. Data highlights the variance between specialized centers (e.g., DD Nord) and regional hubs relative to the 23% national hospital average.

The 2024 analysis of ASM use across hospitals in Saxony reveals significant heterogeneity in prescribing three third generation ASMs, BRV, PER, and CNB out of the total six ASMs analyzed (Figure 4). National benchmark data indicates that general practitioners in Germany (All GP DE) maintain a 5% rate. The utilization of these three more recently approved ASMs exhibits significant regional variance across the Saxon inpatient sector. While Centers in DD-Nord demonstrate a high integration rate of 31%, exceeding the national hospital average of 23%, other regional hospitals in the cohort show substantially lower relative exposure, with rates ranging between 2 and 5%. This highlights a significant disparity in access to more recently approved therapies between specialized centers and general regional inpatient facilities.

In the analyzed Saxon cohort, the proportional utilization of levetiracetam remains higher than that of LTG across all prescriber groups. This distribution reflects the total population of patients under active treatment, including long-term stable cases. While the office-based sector shows the highest volume of both agents, the ratio remains skewed toward levetiracetam, a trend also observed in the general practitioner (OTH) and hospital (AMB) categories.

### 3.4 Regional distribution of the LEV to LTG prescribing ratio across provider groups in Saxony relative to SANAD II benchmarks

**Figure 5:**
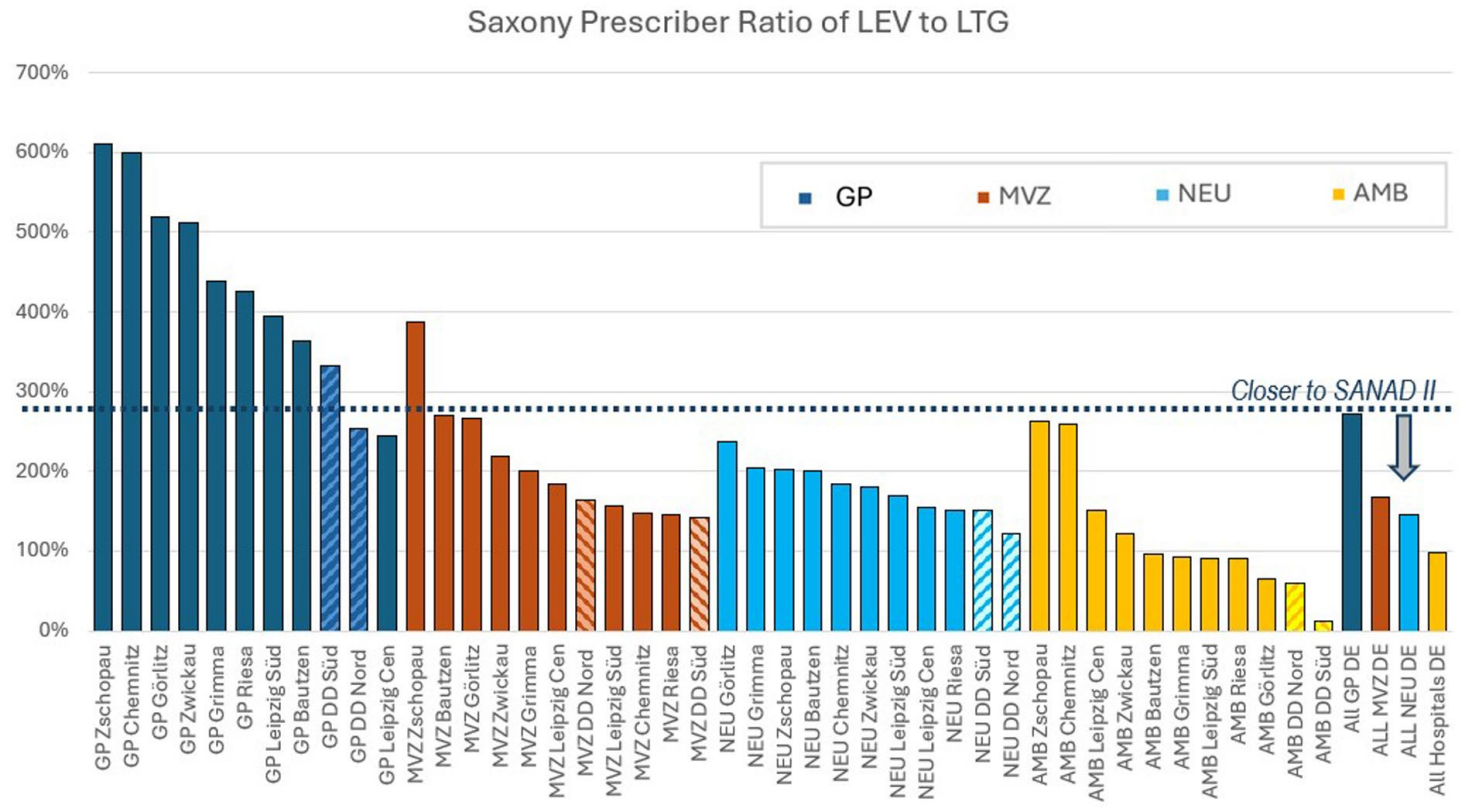
Regional Distribution of the LEV to LTG Prescribing Ratio Across Provider Groups in Saxony Relative to SANAD II Benchmarks: General Practitioners (GP), Outpatient Neurologists (NEU), Medical Care Centers (MVZ), and Specialized Hospital Outpatient Clinics (AMB). Horizontal reference line indicates the national GP benchmark (~300%). Data points illustrate the inverse relationship between medical specialization level and the LEV: LTG ratio, highlighting the shift toward LTG-dominant Closer to SANAD II prescribing in specialized DGfE centers (e.g., DD) compared to rural GP sectors. Striped columns highlight providers located within the Dresden metropolitan area, illustrating a geographic proximity effect where local prescribing patterns align more closely with the specialized DGfE center.

The analysis of the LEV to LTG ratio across Saxony reveals a distributional gradient that correlates with the level of facility specialization (Figure 5). In the primary care sector, particularly within some sub-regions, the LEV to LTG ratio is significantly higher than the national primary care benchmark (300%), with some clusters reaching levels between 500 and 600%. This ratio progressively narrows as the level of neurological specialization increases. Moving from office-based neurologists (NEU) to Medical Care Centers (MVZ), the distribution approaches a 1:1 balance. The lowest ratios in the state are observed in the specialized DGfE hospital outpatient clinics (AMB) in Dresden, where the ratio falls below the 100% mark. This indicates a higher relative utilization of LTG compared to LEV within these specialized settings.

### 3.5 Spatial distribution of outpatient neurologists and longitudinal analysis of specialized referral pathways to the Kleinwachau DGfE Center in Saxony (2022–2024)

**Figure 6:**
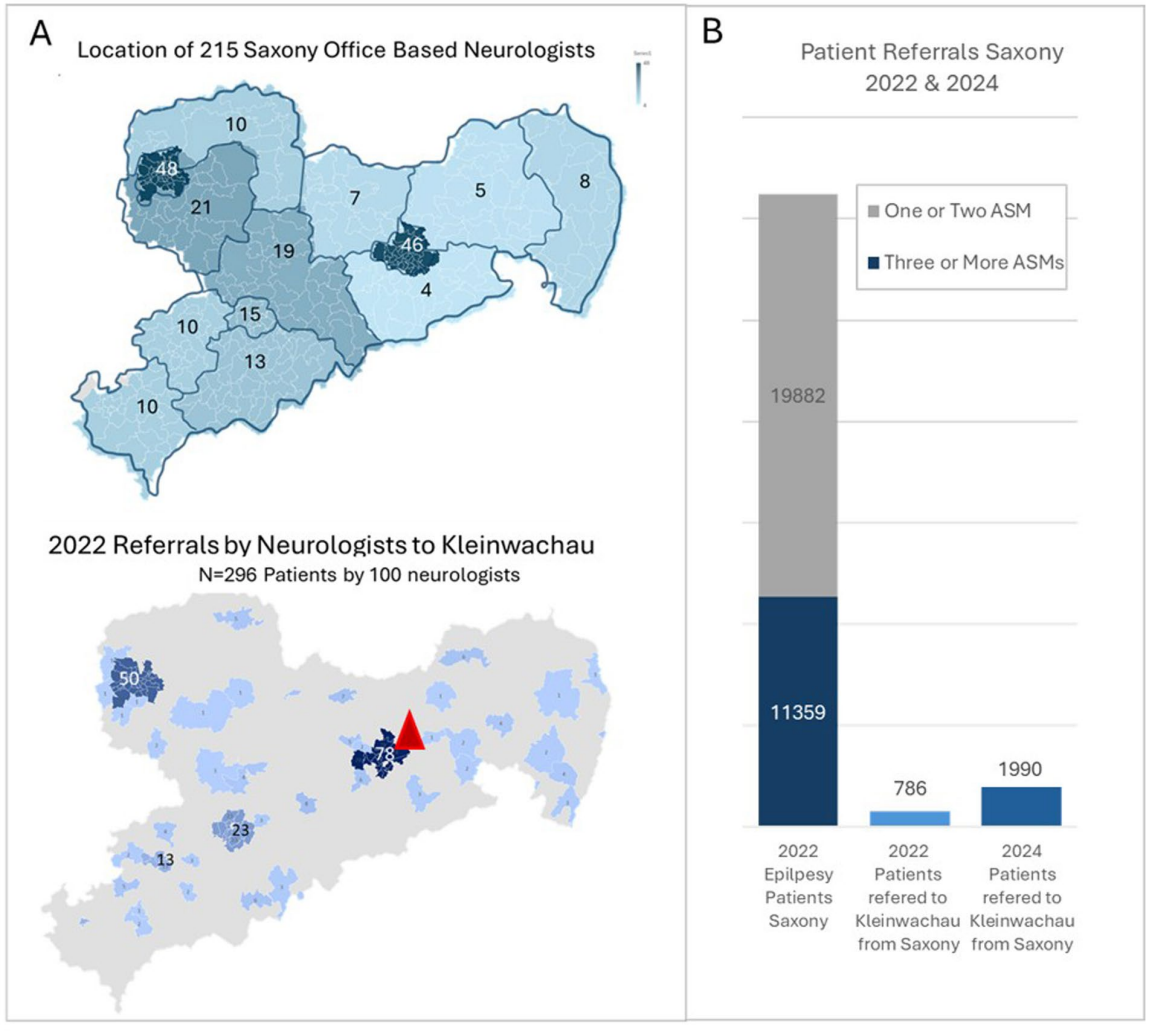
Spatial Distribution of Outpatient Neurologists and Analysis of State Referrals to the Kleinwachau DGfE Center in Saxony (2022–2024). Spatial Distribution of Outpatient Neurologists and Analysis of State Referrals to the Kleinwachau DGfE Center in Saxony (2022-2024). **(A)** Geospatial mapping of *N* = 215 geocoded outpatient neurologists (Sächsische Landesärztekammer (SLÄK) physician finder) showing significant concentration in Leipzig (*n* = 48) and Dresden (*n* = 46). While urban neurologists demonstrate higher referral activity, several districts (indicated in gray) show no recorded referral activity to the center during the study period. **(B)** Referral volume showing an increase in total referred patients from 2022 to 2024. Despite this growth, the total number of referrals remains significantly lower than the estimated regional burden of refractory patients, indicating a persistent gap in access to the state's only DGfE-certified center. Red triangle represents location of Kleinwachau.

Figure 6 provides a geographical and longitudinal analysis of patient movement within the Saxon healthcare system, with a focus on the role of the Kleinwachau DGfE Center. Analysis of regional records indicates a total capacity of approximately 245 outpatient neurologists (comprising both office-based and MVZ-employed specialists) across the state. Using the Sächsische Landesärztekammer (SLÄK) physician finder, 215 individual neurologists were verified by name and address for geocoded spatial analysis. These specialists are heavily concentrated in the urban centers of Leipzig (n = 48) and Dresden (*n* = 46), which together represent nearly 44% of the mapped workforce. Rural areas have therefore lower access to office-based neurological care.

The spatial and clinical data reveal a discrepancy between the estimated drug-resistant epilepsy patient population and observed referral activity. According to IQVIA data, of the 31,241 total epilepsy patients in Saxony in 2022, approximately 36% (*n* = 11,359) tried three or more ASMs (7). Under the delegated care model, where one third of patients are maintained by General Practitioners and 5% are managed in specialized outpatient clinics (AMB), the remaining drug-resistant epilepsy cohort of approximately 7,100 patients is distributed among the 245 outpatient neurologists. Under these assumptions that would correspond to approximately 25–30 drug resistant patients per office-based neurologist.

While the referral network involves a diverse range of healthcare providers (Table 1), Figure 6 specifically focuses on the prescription patterns of outpatient neurologists (NEU and MVZ) as the primary specialists responsible for the long-term management of drug-resistant epilepsy epilepsy. Assessing this cohort independently allows for a clearer evaluation of office-based neurologist referral behavior as well as regional access gaps, independent of other non-specialist providers or the volume within specialized AMB clinics.

Longitudinal data reveals a significant expansion in referral volume, though the composition of referring providers has shifted (Table 1). Between 2022 and 2024, the number of active referring neurologists (NEU and MVZ) grew from 100 to 125, with their patient contributions increasing from 296 to 422. The strongest growth occurred within the DGfE specialized center Kleinwachau, which rose from 69 to 812 total patients (including 296 from the Dresden staff). These figures primarily represent internally managed specialized cases and institutional transfers rather than external referrals, reflecting the center’s role as a high-volume hub for complex diagnostics. Additionally, while the number of referring General Practitioners (GPs) and other providers slightly decreased (175 to 142), their total patient output more than doubled, reaching 522 referrals in 2024.

The high patient volume at the Kleinwachau sites (*n* = 812) reflects its role as a central point for specialized care rather than high referral rates from individual doctors in those regions. While office-based neurologists typically refer a limited number of patients annually (maximum 15), the center serves as a collective destination for specialized diagnostics across the state. This indicates that the center’s growth is driven by the concentration of complex cases from across Saxony rather than resident Dresden patients. Many rural districts, represented by the gray regions in Figure 6A, recorded zero specialist referral activity. These findings indicate that a significant proportion of patients do not access specialized center-based care, a deficit likely driven by geographical distance.

### 3.6 Comparative analysis of regional referral density (2024) and the concentration of pharmacological leadership in epilepsy care in Saxony

**Figure 7:**
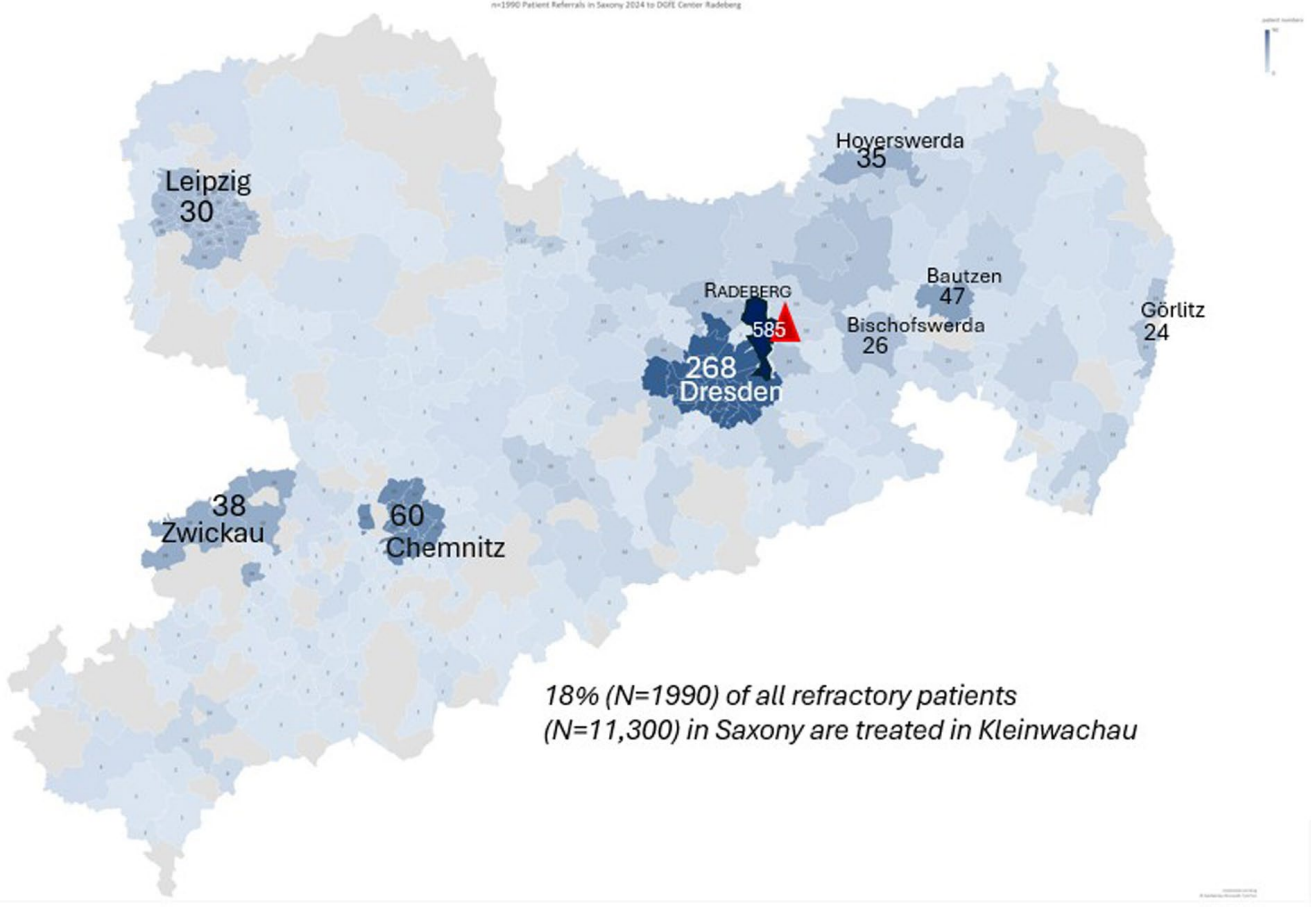
Analysis of Regional Referral Density (2024) to DGfE Center Kleinwachau. The heat map illustrates the spatial distribution of all 2024 in state (*N* = 1,990) epilepsy patients seen at the Kleinwachau DGfE Center. Referral density remains highest within Radeberg (*n* = 585) and Dresden (*n* = 268), while concentrated urban referral activity is visible from Chemnitz (*n* = 60), Bautzen (*n* = 47), Zwickau (*n* = 38), and Hoyerswerda (*n* = 35). Red triangle indicates the location of the Kleinwachau DGfE certified Epilepsy Center.

Spatial analysis of 2024 referral patterns reveals that 1,990 of the estimated 11,359 drug-resistant epilepsy patients in Saxony are managed at the Kleinwachau specialized center; 233 referrals came from outside of the state (Figure 7).

While the network of referring specialists has expanded to 125 office-based neurologists, these practitioners account for 422 of the total referrals. In contrast, a small core of 9 specialized neurologists based at the Kleinwachau, Radeberg and Dresden locations provides care for 812 patients. Geographic heat mapping shows that while referral density is highest in the immediate vicinity of Dresden (*n* = 268) and Radeberg (*n* = 585), significant patient cohorts from rural areas such as Bautzen (*n* = 47) and Chemnitz (*n* = 60) travel to the center. Notably, the Leipzig region shows a lower referral volume (*n* = 30) relative to its high density of 48 office-based neurologists. As a result, only a minority of therapy-difficult patients statewide appear to access specialized center care, indicating regional variation between expected need and observed referral patterns.

### 3.7 Treatment gap advisory board findings

The Treatment Gap Advisory Board (January 2026) identified several structural barriers to care in Saxony, a state of 4 million residents characterized by its two major urban centers, Dresden and Leipzig, and a large rural population. These systemic hurdles, summarized in Table 2, contribute to delayed or suboptimal patient management. A central challenge is the therapeutic lag in transferring patients with drug resistant epilepsy to specialized centers, often because the possibility for clinical improvement through tertiary intervention is unrecognized in primary and secondary care.

Furthermore, the board noted a lack of structured follow up after emergency room visits, often due to the need for better coordination between inpatient and outpatient sectors. This fragmentation can lead to therapeutic inertia; for example, hospital physicians may avoid starting titration regimens such as LTG due to uncertainty regarding outpatient up-titration. Consequently, several regions have similar reports of patients being discharged with suboptimal ASM regimens or remaining on sub-therapeutic doses for several months. This situation appears to contribute to the high prevalence of LEV use in Saxony and can also increase the risk of avoidable emergency readmissions.

To address these issues, the board recommended a multi-level strategy: empowering patients through awareness campaigns, establishing a regional communication network for case consultations, and publishing clear, measurable referral criteria in the *Ärzteblatt* to bridge the gap between outpatient neurologists and specialized epilepsy centers.

## 4 Discussion

Saxony reflects core features of the German epilepsy care system, high reliance on delegated outpatient care, a numerically dominant hospital workforce with limited outpatient prescribing remit, and strong geographic disparities in specialist density that are shared across multiple federal states, particularly in eastern and rural regions. The findings and recommendations therefore provide a lens through which broader structural dynamics of epilepsy care in Germany can be examined.

### 4.1 Structural distribution of epilepsy care and guideline adherence

In Germany, a small set of antiseizure medications, LTG, LEV, and lacosamide, account for the large majority of prescriptions for focal epilepsy. However, previously published national data reveals significant differences between sector specialties: GPs are significantly more likely to prescribe LEV as a first-line therapy, whereas neurologists are nearly 10% more likely to prescribe LTG (29% vs. 20%) (3). In this context this divergence reflects sector-specific prescribing patterns. The persistently higher LEV to LTG rations in certain regions contrast with prescribing profiles typically observed in comparable sectors across Germany, reflecting greater reliance on the perceived favorable pharmacokinetic profile and immediate dosing properties of LEV. This pattern may reflect the practical considerations associated with LTG’ slower titration requirements despite its designation as first line therapy in the current (S2k) guidelines.

The fact that GPs account for approximately one-third of prescriptions reflects the German delegated care model. While neurologists typically initiate complex titration for focal patients, GPs manage long-term maintenance. This is particularly problematic for older patients; despite having a higher prevalence of epilepsy and greater clinical complexity, this demographic is seen more frequently by GPs than neurologists (7). Consequently, office-based neurologists and GPs have become the dual pillars of focal epilepsy care out of necessity, as hospital-based (AMB) clinics function primarily as referral hubs rather than routine providers. However, this study reveals a stark structural imbalance: while 57% of Saxony’s neurologists are based in hospitals, the actual burden of outpatient care—including the management of complex elderly cases—is sustained almost entirely by the remaining 43% of office-based specialists and the primary care network.

The relative contribution of office-based neurologists varies across Saxony, with several regions showing lower neurologist involvement relative to estimated epilepsy burden. This aligns with national findings that while neurologists more frequently initiate LTG as first line therapy, their geographical distribution remains a factor in patient access. This matters clinically, as national adherence data suggest that patients with at least one neurologist contact achieve a 20% relative increase in treatment adherence (84.5% vs. 70.3%) compared to those managed without specialist input (8). This gap identifies a significant clinical disparity: while GP prescribing data confirms that primary care physicians account for a substantial share of ASM exposure across all regions, prescribing patterns demonstrate divergence from first-line recommendations in certain regions. This is evidenced by the persistence of high LEV-to-LTG ratios and the limited utilization of third-generation ASMs in the primary care sector, reflecting persistence of established prescribing patterns alongside slower uptake of newer therapeutic options.

### 4.2 Role of epilepsy centers and use of newer ASMs

We have also uncovered a misalignment between structural capacity and clinical practice in the Saxon hospital landscape. Workforce data shows that 63% of neurologists are hospital-based, and although care is maintained by outpatient doctors (GP, MVZ and office-based Neurologists) the 2024 prescribing data suggests that the DGfE center in DD Nord accounts for a disproportionally high share of third generation ASMs use. Although office-based neurology drives overall volume, the uniquely high uptake of third-generation ASMs at DD Nord (31%) reflects the localization of a DGfE-certified specialized center. These data are consistent with a high but small concentration of epilepsy-specialized neurologists who have extensive experience functioning as regional lighthouses. These epileptologists treat difficult epilepsy patients, prescribe new ASMs and send the newly adjusted patients back to their outpatient physicians. Together with their regular educational seminars and EEG courses, they work to spread epilepsy knowledge, including about new ASMs and more guideline conform practices. While the concentration of third-generation ASM uptake within DD Nord aligns with its formal role as a DGfE certified center responsible for regional training and escalation support, University based outpatient clinics with an *Ambulanz* likewise carry a mandate for epileptological leadership. The persistence of marked sectoral geographic gradients suggests that diffusion of escalation competence across the broader ambulatory network remains incomplete.

### 4.3 Silent refractoriness, referral behavior and specialization thresholds

Our results demonstrate that the level of medical specialization is a primary determinant of adherence to international evidence-based standards. While the high LEV-to-LTG ratios among rural GPs suggest a reliance on levetiracetam—likely due to its ease of titration and perceived safety—this trend persists despite growing evidence of neuropsychiatric adverse effects (11). In contrast, hospital outpatient providers (AMB) exhibit prescribing patterns more closely aligned with first line recommendations. Specifically, AMB facilities in Dresden achieve ratios most consistent with SANAD II guidelines, underscoring the vital role of specialized expertise in early therapeutic management. Within the German healthcare framework, this divergence emphasizes that specialist involvement is crucial for optimizing first line treatment selection.

Our findings reveal a preference for levetiracetam over LTGthat persists across all sectors. This is noteworthy given that head-to-head trials have established LTG as the more effective and better-tolerated first-line option for focal epilepsy (12). Current German clinical practice guidelines also reflect this preference (32). However, the high prevalence of levetiracetam in our data likely reflects to some extent legacy prescribing patterns (*Bestandsfälle*) since clinicians are hesitant to switch medications in patients perceived as having good seizure control. Although some of the observed patterns likely reflect legacy maintenance rather than current initiation decisions, non-specialists may overlook potential side effects resulting from long-term use of sub-optimal ASM combinations (13).

Our data analysis suggests that therapy-difficult epilepsy is expected to represent a recurrent feature of routine neurological practice across Saxony even after accounting for GP maintained care. This structural expectation is further supported by independent health insurance data that provide an external perspective on the overall medication burden of epilepsy within the state. Independent morbidity data from the BARMER Institute for Health Systems Research (14) show that Saxony is characterized not only by elevated epilepsy prevalence but also by the highest Defined Daily Dose deviation nationwide (+11%), alongside Thuringia, as well as a + 6% deviation in the number of distinct prescribed active substances per affected person. Although the higher proportion of elderly patients in Eastern Germany likely contributes to increased medication exposure and multimorbidity, the contrast with demographically comparable states such as Brandenburg suggests that age structure alone does not fully explain the magnitude of pharmacological intensity observed in Saxony. The convergence of elevated prevalence increased medication burden, and substantial polypharmacy indicates a population-level complexity that places particular demands on coordinated escalation pathways and specialist integration (7).

Taken together, the BARMER data and the 2024 referral density suggests an ongoing state of *silent refractoriness* where a significant portion of therapy-difficult patients may not be accessing escalation pathwaysWhile the increase from 100 referring neurologists in 2022 to 125 in 2024 indicates a broadening network, it also highlights that a sizable share of the state’s neurological workforce may not be consistently connected to formal escalation pathways. This lack of systemic connectivity is often compounded by individual clinical decision-making processes, where the threshold for recognizing the need for escalation remains unexpectedly high (13, 15).

Evidence from studies showing the slow uptake of LTG as well as more recent European virtual patient simulation studies are consistent with the presence of diagnostic and therapeutic inertia in epilepsy care (16, 17). In the series of MEDSCAPE practice cases, neurologists from across the EU demonstrated low rates of drug-resistant epilepsy recognition and delayed escalation of therapy, despite access to guideline-consistent information, indicating that knowledge gaps and decision thresholds remain significant barriers to timely referral and treatment optimization (18–20). Beyond guideline adherence, emerging evidence indicates that recurrent seizures may contribute to cumulative cellular network and structural changes as well as long term individual and societal burden demonstrating the importance of timely optimization of therapy (15).

### 4.4 The clinical cost of the LEV-preference

The structural over-reliance on LEV carries significant clinical implications that extend beyond simple guideline non-adherence (13). While the ease of titration makes LEV an attractive default for primary care and time-constrained outpatient specialists, this prescribing convenience may come at the expense of patient neuropsychiatric health. Large-scale evidence indicates that nearly one-fifth of patients will experience some form of neuropsychiatric adverse reaction to LEV (21). Recent clinical data quantifies this burden, showing that 13.3% of adults develop symptoms of depression or emotional lability, while approximately 1 to 1.4% progress to full psychosis—a significantly higher risk than that associated with other ASMs (21, 22). These side effects are not merely clinical nuisances; they represent a substantial economic burden among ASM-related adverse reactions and are a primary driver of treatment discontinuation.

The SANAD II trial established that LEV is inferior to LTG regarding treatment failure, primarily due to a poorer tolerability profile (12). These data formed the foundation for updated German guidelines (32). Furthermore, recent analyses suggest that newer SV2A modulators such as brivaracetam (BRV) offer a superior psychobehavioral profile compared to LEV (23). This shift is already reflected in the usage patterns of specialized AMB facilities in Dresden Nord and other German hospital-based outpatient centers. By maintaining LEV as a first line default, the current care model in Saxony may expose patients to avoidable psychiatric comorbidities. This creates a cycle of treatment failure where discontinuation is driven by intolerable side effects rather than a lack of seizure control. This persistent clinical reliance on LEV often stems from an educational gap, where unfamiliarity with managing LTG’s prolonged titration schedule and potential hypersensitivity risks acts as a barrier to guideline implementation.

The persistence of this suboptimal prescribing pattern is likely rooted in the structural and economic landscape of the German healthcare system. Financial pressures, such as the *Wirtschaftlichkeitsgebot* (§ 12 SGB V), may explain some resistance to newer, more expensive ASMs (24). However, these constraints do not justify the preference for LEV over the similarly priced LTG. Beyond financial regulations, the concentration of third generation prescribing in specialized hubs like DD Nord suggests that the gap is also one of expertise distribution. This indicates that structural opportunities for specialized training in rational drug combinations must be better distributed across the broader neurologist population. Ultimately, the fact that the 2024 referral volume reached 1,990 patients—a significant increase from the 2022 cohort—suggests that specialized centers are now functioning as essential safety valves. They are managing the overflow for a system where routine specialist care has reached its limit in handling complex, drug-resistant cases.

### 4.5 Centralization capacity constraints and geographic access

The 2024 Kleinwachau referral data confirms a significant concentration of clinical responsibility within a single institution. Currently, the neurology team at Kleinwachau manages a greater volume of ASM adjustments than the combined total of the 125 referring neurologists across the state. This centralization ensures high quality care for those within the center’s immediate reach, yet it also exposes a stark geographic imbalance in specialist networking.

Leipzig represents a primary urban center with a high density of neurology practices, yet it currently exhibits a lower referral volume compared to the eastern districts. This is particularly noteworthy given that statewide morbidity data (BARMER analysis) indicates that the rural areas surrounding Leipzig represent some of the highest epilepsy burdens in the state. This discrepancy points toward a structural opportunity to better integrate western Saxony into specialized referral pathways.

The reliance on a single hub for nearly 2,000 patients suggests a system operating at its maximum capacity. To improve epilepsy care pathways in the state, the current model of pharmacological leadership should evolve toward a more distributed network. This could be achieved through a combination of expanded hospital-based outpatient authorizations (*Ermächtigungen*) and enhanced, structured coordination between specialized centers and outpatient neurologists. Such a model would ensure that specialized evidence-based care is more easily accessible to patients in the western region, regardless of their proximity to a single central hub.

### 4.6 Role of leadership

At the system level, the most consequential finding is the distance between pharmacological expertise and longitudinal care in Saxony. Across multiple, independent indicators, first-line LEV/LTG ratios, adoption of newer ASMs, and referral density to specialized centers, the data consistently show that treatment escalation competence is concentrated within a very small number of highly specialized clinicians, while the majority of epilepsy care volume is carried by general practitioners and office-based neurologists who operate under structural constraints that limit the initiation or maintenance of advanced therapeutic strategies. This is consistent with a structural pattern in which responsibility for long-term management is widely distributed while epileptology expertise is more centralized. The result is a large intermediary space in which therapy-difficult patients remain in ongoing care without systematic progression to third-line treatments or specialized evaluation.

This treatment gap is unlikely to be resolved by expanding a single center’s capacity; rather, it points to a need for better alignment between who manages epilepsy patients day-to-day and who is structurally enabled to lead evidence-based escalation, with implications for training, outpatient care, and regional responsibility-sharing in epilepsy patient pathways.

### 4.7 Academic positioning of epilepsy and its implications for care

One possible explanatory, piece is the historically limited positioning of epilepsy as a flagship research disease within Saxony’s two major university clinics (Dresden and Leipzig), which contrasts with German states that host dedicated epileptology departments and long-standing epilepsy research programs (e.g., Bonn/Freiburg), and may may partially relate to why epilepsy expertise in Saxony is more concentrated outside the university hubs: a scoping PubMed affiliation scan shows that Dresden- and Leipzig-affiliated epilepsy-related outputs are present but often clustered in adjacent domains (e.g., seizure forecasting/neurosurgery/engineering collaborations in Dresden and status epilepticus/acute care and genetics-oriented work in Leipzig), rather than a sustained volume of clinically anchored epileptology research that typically builds referral networks, trial participation, and early adoption norms, positioning Kleinwachau (a non-university institution) as a key regional driver of clinical innovation, where specialized research is directly integrated into the state’s most active escalation hub.

That gap is further sharpened when the publication landscape is examined more closely: a substantial proportion of epilepsy-related outputs affiliated with Leipzig originate from human genetics groups that focus on molecular mechanisms, variant discovery, or syndromic characterization, but do not provide epilepsy treatment themselves and show limited structural integration with clinical epileptology or outpatient care pathways. While this work is scientifically valuable, it does not generate the downstream effects typically associated with epilepsy as a research disease, such as clinical trial infrastructure, treatment algorithms, escalation norms, or durable referral networks, and therefore may have fewer direct downstream effects on pharmacological leadership within the university clinics themselves.

In practical terms, Saxony’s apparent epilepsy “research activity” is partially decoupled from epilepsy care, consistent with a specialized ecosystem where advanced clinical leadership is anchored in non-university centers such as Kleinwachau, which play a vital role in translating academic insights into routine drug-resistant epilepsy care.

The structural de-prioritization of epilepsy in Saxony can be understood as a legacy of the historical divergence between the “academic approach” of university neurology and the “deacon approach” of specialized institutions (25). Historically, German epileptology developed within specialized colonies such as Bethel and Kleinwachau, which provided comprehensive longitudinal care outside the traditional university system (26). While other regions have bridged this gap through endowed professorships and integrated university departments, Saxony’s current care model still reflects this historical separation (25). The resulting absence of a dedicated academic anchor at the university level reinforces a system where epilepsy is managed as a condition of routine care rather than a flagship for institutional innovation, ultimately contributing to the care plateau observed in our data, where patients with difficult-to-treat epilepsy remain in a cycle of maintenance therapy without systematic progression to evidence-based escalation or specialized evaluation.

This institutional gap is further compounded by the positioning of epilepsy within academic research. Despite its high prevalence and burden, epilepsy has long been described as a condition subject to persistent social and medical stigma, including within high-income countries, where it is often framed as a chronic management problem rather than a locus of biomedical innovation (27, 28). Scientometric analyses of epilepsy research have shown that sustained clinical–academic leadership and institutional concentration are key drivers of visibility, innovation, and guideline translation, yet epilepsy research output in Germany has historically been diffuse and unevenly anchored across institutions relative to disease burden (29, 30).

In this context, stigma operates not as overt exclusion but as structural de-prioritization: epilepsy remains present in academic output yet insufficiently positioned as a flagship research field capable of attracting sustained institutional investment, which may contribute to the separation the between routine care, pharmacological leadership, and innovation observed in the present analysis.

#### 4.7.1 External validity and global relevance

While the structural nuances and funding mechanisms described here are specific to the German healthcare framework, the underlying dynamics of these prescription gaps and referral barriers have broad external validity for other high-income countries. International literature consistently confirms that across various health systems, the transition of patients from routine, community-based management to specialized tertiary epileptology remains a universal bottleneck characterized by widespread therapeutic inertia (13, 31). The under-recognition of drug resistance by general providers and a persistent failure to implement breakthrough treatments are global challenges, rather than regional anomalies (5). Whether navigating insurance-driven models or European centralized systems, the concentration of advanced pharmacological leadership within limited specialized hubs creates clinical care plateaus that transcend national borders (17). Consequently, our findings regarding the separation between academic research and routine clinical delivery offer valuable insights for international health systems seeking to optimize specialized pathways and reduce the time-to-referral globally.

#### 4.7.2 Limitations: geographic data

The prescribing volumes for the Dresden and Kleinwachau AMBs likely introduces a geographic distortion. Because the data is anchored to the provider’s address, the maps may over-represent the urban drug-resistant epilepsy population while under-representing the rural ‘outland’ districts where these patients actually reside. Future studies utilizing patient postal codes (PLZ) would be required to precisely map the true distance-to-care for this cohort.

## 5 Conclusion

The 2026 Saxony Expert Panel confirms that the dissemination of clinical guidelines is not enough to bridge the knowledge and expectation gaps within the German outpatient sector. The structural hurdles identified in Saxony demonstrate that a shift from passive information sharing to active regional networking is essential. By implementing the proposed *red flag catalog* and establishing dedicated communication networks, the delay to specialized intervention for pharmacoresistant patients can be shortened.

The data suggests that for rural and distant regions to benefit from specialized expertise, the physical presence of a center must be supplemented by a structured, bi-directional referral framework. This study highlights that specialized centers are most effective when they function as accessible knowledge hubs for referring physicians in the more rural districts. By measuring local outcomes against established standards, the system can move away from silent refractoriness toward a transparent and data driven model of care. Ultimately, these findings offer a scalable framework for other German federal states to address the divide between primary care and specialized epilepsy centers.

## Data Availability

The data analyzed in this study is subject to the following licenses/restrictions: Data sets were generated by IQVIA and data used in this paper is available on resonable request. The databases contain solely anonymous data and do not identify any personal information, in compliance with §3 Abs. 6 of the German Federal Data Protection Act (Bundesdatenschutzgesetz). Identification of individual patients, healthcare practices and clinics was not possible; therefore, informed consent was not required. Requests to access these datasets should be directed to sonya.faber@angelinipharma.com.

## References

[1] Willems LM Hochbaum M Zöllner JP Schulz J Menzler K Langenbruch L . Trends in resource utilization and cost of illness in patients with active epilepsy in Germany from 2003 to 2020. Epilepsia. (2022) 63:1591–602. doi: 10.1111/epi.17229 35305026

[2] Willems LM Hochbaum M Frey K Schulz J Menzler K Langenbruch L . Multicenter, cross-sectional study of the costs of illness and cost-driving factors in adult patients with epilepsy. Epilepsia. (2022) 63:904–18. doi: 10.1111/epi.17174 35192210

[3] Strzelczyk A Kostev K Margraf NG Schulze-Bonhage A. Initial monotherapy and further anti-seizure medication regimens in adult patients with epilepsy: A population-based, longitudinal, retrospective analysis of German prescription data. Neurol Ther. (2026) 1:900. doi: 10.1007/s40120-026-00900-8 PMC13172149 41817647

[4] Kwan P Arzimanoglou A Berg AT Brodie MJ Hauser WA Mathern G . Definition of drug resistant epilepsy: consensus proposal by the ad hoc task force of the ILAE commission on therapeutic strategies. Epilepsia. (2010) 51:1069–77. doi: 10.1111/j.1528-1167.2009.02397.x 19889013

[5] Klein P Krauss GL Steinhoff BJ Devinsky O Sperling MR. Failure to use new breakthrough treatments for epilepsy. Epilepsia. (2023) 64:1458–65. doi: 10.1111/epi.17564 36855241

[6] Bundesärztekammer. *Ergebnisse der Ärztestatistik zum 31.12.2023*. (2024). Available online at: https://www.bundesaerztekammer.de/baek/ueber-uns/aerztestatistik/2023.

[7] Strzelczyk A Margraf NG Faber SC Fulgeri N Schulze-Bonhage A. Demographics and care of epilepsy in older adults in Germany. Seizure: European. J Epilepsy. (2025) 128:4–15. doi: 10.1016/j.seizure.2025.02.003 40050190

[8] Margraf NG Strzelczyk A Kostev K Pérez Rosal SR Greshake B Von Podewils F . Retrospective observational study of treatment and referral pathways of patients with epilepsy in Germany. Seizure. (2026) 138:157–66. doi: 10.1016/j.seizure.2026.04.001, 42048773

[9] Strzelczyk A Bergmann A Biermann V Braune S Dieterle L Forth B . Guideline adherence and treatment gaps in focal epilepsy care: A national analysis of the German primary and specialist sectors. J Neurol. (2016) 1:11. doi: 10.1016/j.yebeh.2016.07.037

[10] Strzelczyk A Griebel C Lux W Rosenow F Reese JP. The burden of severely drug-refractory epilepsy: A comparative longitudinal evaluation of mortality, morbidity, resource use, and cost using German health insurance data. Front Neurol. (2017) 8:712. doi: 10.3389/fneur.2017.00712 29312132 PMC5743903

[11] Bermeo-Ovalle A. SANAD II: dear Levetiracetam, the honeymoon is over. Epilepsy Curr. (2021) 22:18–21. doi: 10.1177/15357597211052129 35233190 PMC8832349

[12] Marson A Burnside G Appleton R Smith D Leach JP Sills GJ . The SANAD II study of the effectiveness and cost-effectiveness of levetiracetam, zonisamide, or lamotrigine for newly diagnosed focal epilepsy: an open-label, non-inferiority, multicentre, randomised controlled trial. Lancet. (2021) 397:1361–74. doi: 10.1016/S0140-6736(21)00247-6 PMC8047799 33838757

[13] Penovich PE Stern JM Becker DA Long L Santilli N McGuire L . Epilepsy treatment complacency in patients, caregivers, and health care professionals. Neurol Clin Pract. (2021) 11:377–84. doi: 10.1212/CPJ.0000000000001066 34824892 PMC8610521

[14] BARMER Institut für Gesundheitssystemforschung. *Epilepsie: Morbiditäts- und Sozialatlas*. Versorgungskompass. (2023). Available online at: https://www.bifg.de/atlas/epilepsie.

[15] Walker MC Galovic M Álvarez-Barón E Strzelczyk A. Seizures beget more than seizures: understanding the cellular, structural, individual and societal impact of seizures in epilepsy. Epilepsia open. (2025) 10:1762–73. doi: 10.1002/epi4.70143 41071564 PMC12716293

[16] Compagno Strandberg M Söderberg-Löfdal K Kimland E Dahlin M Källén K. Evidence-based anti-seizure monotherapy in newly diagnosed epilepsy: A new approach. Acta Neurol Scand. (2020) 142:323–32. doi: 10.1111/ane.13312 32627842

[17] Poza JJ Gobbo M Palanca Cámara M Pérez-Domper P Aledo-Serrano Á. Key steps and barriers in the journey of patients with epilepsy through the National Healthcare System in Spain: the EPIPASS qualitative study. Epilepsia open. (2024) 9:1731–44. doi: 10.1002/epi4.12984 38965814 PMC11450596

[18] Thevathasan L . Virtual patient simulation improves diagnosis and management but uncovers low drug-resistant epilepsy diagnosis rates. Eur J Neurol. (2025) 32:396.

[19] Thevathasan L Rohani-Montez C Carpenter K DiAmecio M Gupta S Barrios J . *Virtual Patient Simulation Improves Diagnosis and Management but Uncovers Low Drug-Resistant Epilepsy Diagnosis Rates [Poster Presentation]*. 11th European Academy of Neurology (EAN) Congress, Helsinki, Finland. Poster EPO-396. Sponsored by Medscape, LLC. (2025).

[20] Thevathasan L Rohani-Montez CM Carpenter K Scott Smith C Lattanzi S. *Uncovering Knowledge and Practice Gaps in Focal Epilepsy Amongst European Neurologists [Poster Presentation]*. *11th European Academy of Neurology (EAN) Congress*, Helsinki, Finland. Poster EPO-403. Sponsored by Medscape, LLC. (2025).

[21] Campbell C McCormack M Patel S Stapleton C Bobbili D Krause R . A pharmacogenomic assessment of psychiatric adverse drug reactions to levetiracetam. Epilepsia. (2022) 63:1563–70. doi: 10.1111/epi.17228 35298028 PMC9321556

[22] Durairaj T Porchezhian M Kumar M Britto JRJ Saravanan AKM. Beyond seizure control: A case series on Levetiracetam associated psychiatric manifestations. Indian. J Psychol Med. (2025) 15:63453. doi: 10.1177/02537176251363453 PMC12357825 40832649

[23] Willems LM Van der Goten M Von Podewils F Knake S Kovac S Zöllner JP . Adverse event profiles of antiseizure medications and the impact of coadministration on drug tolerability in adults with epilepsy. CNS Drugs. (2023) 37:531–44. doi: 10.1007/s40263-023-01013-8 37271775 PMC10239658

[24] Willems LM Hamer HM Knake S Rosenow F Reese JP Strzelczyk A. General trends in prices and prescription patterns of anticonvulsants in Germany between 2000 and 2017: analysis of national and cohort-based data. Appl Health Econ Health Policy. (2019) 17:707–22. doi: 10.1007/s40258-019-00486-w 31161366

[25] Grewe P Bien CG. 150th anniversary of the Bethel epilepsy center in Germany: an important milestone in the evolution of epilepsy care. Seizure. (2017) 53:110–3. doi: 10.1016/j.seizure.2017.11.007 29173941

[26] Wolf P. Zur Kulturgeschichte der Epilepsie. Epileptologie. (2014) 31:108–19.

[27] Jacoby A Snape D Baker GA. Epilepsy and social identity: the stigma of a chronic neurological disorder. Lancet Neurol. (2005) 4:171–8. doi: 10.1016/S1474-4422(05)70020-X 15721827

[28] Thorbecke R Pfäfflin M. Perception of epilepsy in Germany: results of a population-based survey. Epilepsy Behav. (2022) 128:108556. doi: 10.1016/j.yebeh.2022.108556

[29] Löscher W Schmidt D. New horizons in the development of antiepileptic drugs: innovative strategies. Epilepsia. (2002) 43:1127–34. doi: 10.1046/j.1528-1157.2002.30902.x PMC1574365 16835945

[30] Sander JW. The epidemiology of epilepsy revisited. Curr Opin Neurol. (2003) 16:165–70. doi: 10.1097/00019052-200304000-00008 12644744

[31] Thevathasan L Rohani-Montez C Montez M Carpenter K DiAmecio M Gupta S . *Focal Epilepsy Virtual Patient Simulation Improves Performance in Diagnosis and Management, but Uncovers Inertia [Poster Presentation]*. 11th European Academy of Neurology (EAN) Congress, Helsinki, Finland. Sponsored by Medscape, LLC. (2025).

[32] Holtkamp M May T. W. Berkenfeld R Bien CG Coban I Knake S . Erster epileptischer Anfall und Epilepsien im Erwachsenenalter, S2k-Leitlinie. In Deutsche Gesellschaft für Neurologie (Ed.), Leitlinien für Diagnostik und Therapie in der Neurologie. (2023). Available online at: www.dgn.org/leitlinien (Accessed August 01, 2024).

